# ATR Analysis: Quantifying Molecular and Bulk Responses in Guest–Host Poled Polymers with Push–Pull Heptamethine Chromophores

**DOI:** 10.1021/acs.jpcb.6c03666

**Published:** 2026-08-29

**Authors:** Danning Lyu, Di Zhang, Jingdong Luo

**Affiliations:** † Department of Chemistry, 53025 City University of Hong Kong, Hong Kong SAR, China; ‡ Shenzhen Research Institute, City University of Hong Kong, Shenzhen 518057, China; § State Key Laboratory of Flexible Electronics (LoFE) & Institute of Advanced Materials (IAM), School of Flexible Electronics (Future Technologies), Nanjing Tech University (NanjingTech), Nanjing 211816, China

## Abstract

This study reports a systematic experimental and analytical workflow for evaluating push–pull heptamethine chromophores (PPH-phores) and their poled guest–host polymers for electro-optic (EO) applications. Attenuated total reflection (ATR) spectroscopy was used to accurately measure the optical birefringence and the DC/AC EO coefficients (*r*_33_ values) of poled films, establishing a direct correlation between molecular properties and bulk material responses. Sellmeier analysis of refractive-index dispersion quantified the nonresonant and low-energy resonance contributions and, combined with Kuzyk’s two-component model, enabled reliable extraction of key parameters, including the order parameter (⟨*P*_2_⟩), dipole moment, and linear polarizability of PPH-phores. Incorporating the experimentally determined ⟨*P*_2_⟩, the rigid oriented gas model was applied to derive the first hyperpolarizability (*β*) of PPH-phore directly from measured *r*_33_ values. Across a comprehensive evaluation of 15 PPH-phores and 20 guest–host polymers, we identified an ultralarge dressed *β* of up to −34,000 × 10^–30^ esu and a linear polarizability of up to 9.31 × 10^–22^ cm^3^, the highest values reported to date for PPH-phores, which collectively translate into large *r*_33_ values in poled polymers. This integrated workflow accelerates structure–property relationship studies and facilitates the discovery and optimization of PPH-phores, supporting the scalable development of high-performance organic EO materials.

## Graphical abstract

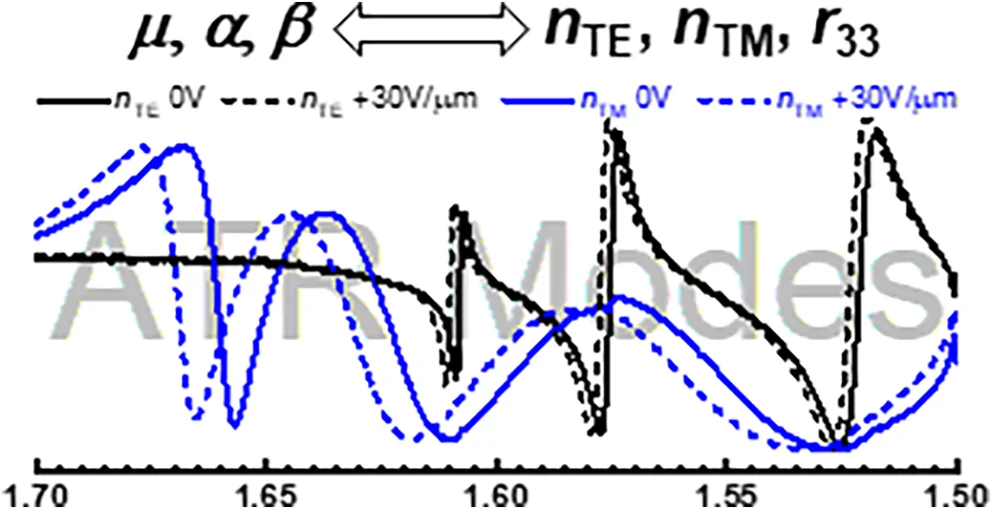

## 1 Introduction

Over the past decade, push–pull heptamethine chromophores (PPH-phores) and their poled polymers have advanced rapidly for electro-optic (EO) applications, keeping pace with the massive growth in the global communication infrastructure, cloud services, and AI workloads.^1–15^ The design, synthesis, and characterization of these organic EO materials follow a multistep pipeline, including modular synthesis of PPH-phores, electric-field poling, and precise optical evaluation of poled polymer films using attenuated total reflection (ATR) spectroscopy (Figure 1).^13,15^

**Figure 1:**
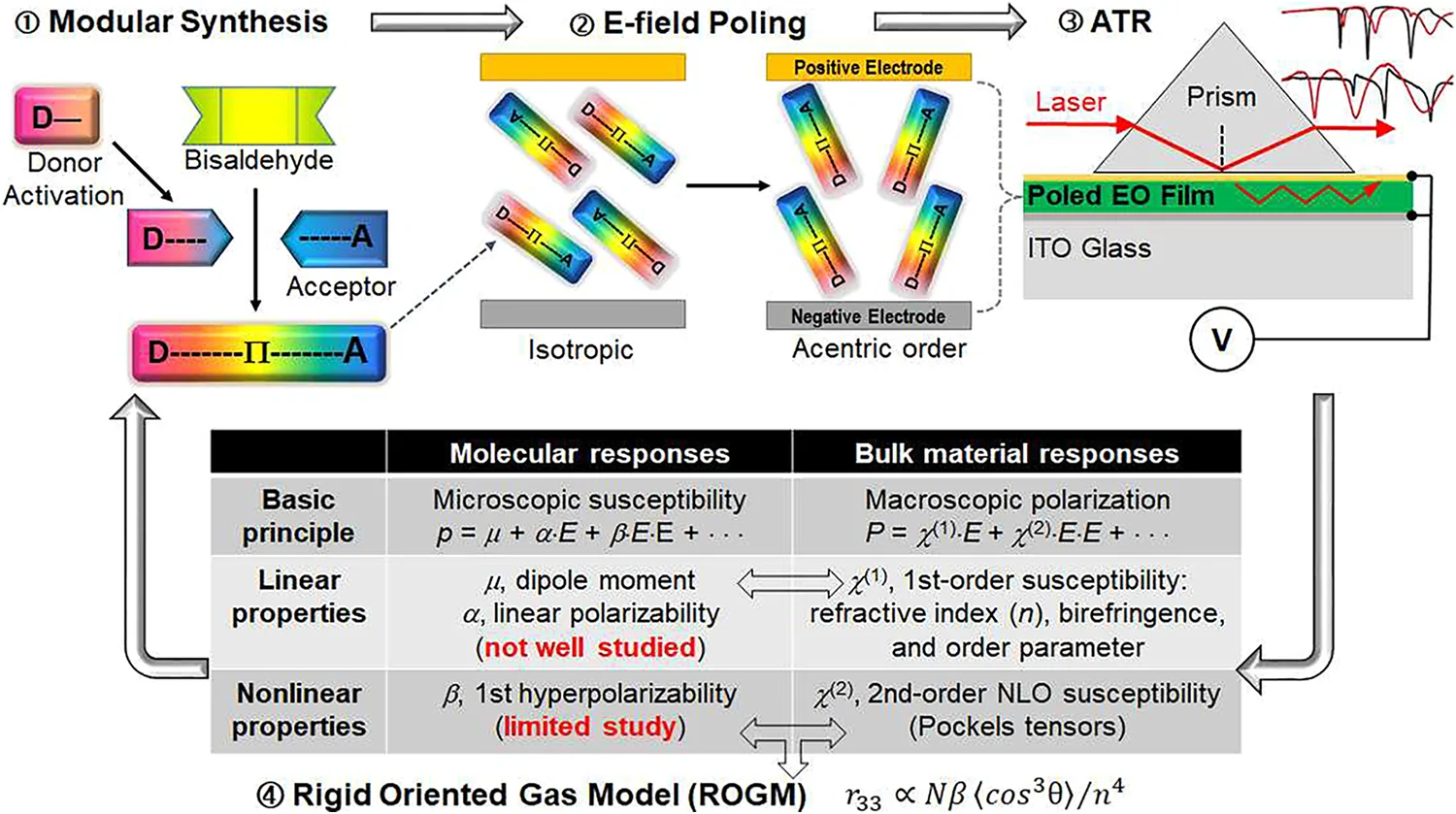
Integrated workflow and fundamental principles for organic EO materials based on PPH-phores: (1) Modular synthesis of Donor-Π-Bridge-Acceptor (D-Π-A) chromophores; (2) E-field Poling of guest–host polymers to induce acentric order from an isotropic state; (3) ATR Spectroscopy for optical characterization of poled films; (4) Rigid Oriented Gas Model (ROGM), which bridges molecular responses (*μ*, *α*, *β*) and bulk optical refractive indices and EO coefficients.^16–21^ Red text indicates current research gaps in understanding the relationship between molecular-level properties and macroscopic responses.

Despite substantial progress, a fundamental gap persists between molecular properties and bulk material performance. While the Rigid Oriented Gas Model (ROGM) defines macroscopic Pockels coefficients (*r*_33_ values) of poled polymers as a function of chromophore’s first hyperpolarizability (*β*), poling-induced polar order, and loading density (*N*),^16–21^ several critical research challenges continue to hinder the systematic establishment of quantitative structure–property relationships:

(1) The “Linear” Knowledge Gap: the macroscopic linear optical properties associated with *χ* ^(1)^, such as refractive index and birefringence, are under-documented, and the underlying molecular dipole moment (*μ*) and linear polarizability (*α*) are poorly understood.^10,11,13,15^ This lack of fundamental data limits our ability to predict how molecular structure dictates the refractive index and optical anisotropy of poled EO films.
(2) Bridging Microscopic Order and Bulk Efficiency: A major hurdle lies in translating isotropic molecular distributions into acentric order through E-field poling. This process is governed by the complex interplay among molecular *μ*, *α*, and intermolecular forces, factors that need to be rigorously examined by the order parameter and ROGM analyses.
(3) Limited Understanding of Molecular Optical Nonlinearity: There is currently limited study on the first hyperpolarizability (*β*) of PPH-phores in poled polymers.^10,11,13,15^ Without a comprehensive library of *β* values for diverse “Donor-Π-Bridge-Acceptor” (D-Π-A) architectures, optimizing the EO responses of poled polymers remains largely an empirical endeavor rather than a quantitative design process.

Addressing these gaps is essential to establish the structure–property relationships between molecular-level properties (*μ*, *α*, *β*) and macroscopic responses. In this work, we present a high-throughput experimental and analytical framework to evaluate PPH-phores and their poled guest–host polymers for EO applications. The modular synthesis of 15 PPH-phores enabled rapid screening of donor–acceptor combinations and *meso*-substitutions. High-precision prism-coupled ATR spectroscopy was used to measure the poling-induced optical birefringence and EO coefficients in poled polymer films. Sellmeier dispersion model and ROGM were then applied to determine the order parameter and molecular *μ*, *α*, and *β* values. This self-consistent approach improves the quantitative analysis of new organic EO materials based on PPH-phores and provides high-quality data sets for future machine-learning-driven molecular discovery.

## 2 Materials and Methods

### 2.1 Poling and Measurement of Refractive Indices and Modulated ATR Spectra for Guest–Host Films

Guest–host polymers were prepared by dissolving the chromophore at a given loading density *N* with poly­(styrene-*co*-methyl methacrylate) (P­(S-*co*-MMA)) using dibromomethane (DBM) as solvent. The *N* is calculated by assuming the composite density of 1.10 g/mL. The formulated solutions were filtered through a 0.2 μm PTFE filter, then spin-coated onto indium tin oxide (ITO) glass substrates. Films were soft-baked, followed by overnight annealing in a vacuum oven at 75 °C to remove residual solvent. Film thicknesses were measured using a DektakXT Stylus Profiler and verified via subsequent ATR measurements.^10,11,13^

A ∼ 10 nm semitransparent gold layer was then sputtered onto the films using a Desk V HP Sputter Unit (Denton Vacuum LLC) to serve as the top electrode for contact poling and ATR-based electro-optic (EO) measurements. The prepared films were poled on a processor-controlled Mettler FP82 hot stage. A typical field of 100 V/μm was applied at 30 °C and the temperature was ramped at 10 °C/min, with leakage current (LTC) monitored in situ using a Keithley 2657A Source-Meter Unit. Optimal poling temperatures (110 to 115 °C) were identified from the onset of a rapid increase in LTC, which occurred near the glass transition temperature (*T*_g_) of guest–host polymers. After poling, the poled films were cooled to the ambient temperature and the voltages were then reduced to zero. A complete poling process for a single poled film typically takes 20–25 min.

After electric-field poling, the reflectivity spectra of the poled films were measured via ATR in a slab waveguide geometry using Metricon 2010/M at 1304 and 1541 nm. The refractive indices *n*_TE_ and *n*_TM_, corresponding to transverse electric (TE) and transverse magnetic (TM) polarizations, were determined. The modulated reflectivity spectra of the poled films were recorded under DC or AC modulation voltages.

All ATR measurements were performed at a near-constant temperature of around 20 °C, with each measurement typically taking 10–25 s. The standard prisms were further calibrated with the Index Calibration Standard (ICS) from Metricon Corp to improve the absolute index accuracy of ±0.0001–0.0002, together with the index resolution of ±0.0003, thickness accuracy of ±(0.5% + 5 nm), and thickness resolution of ±0.3%. In this study, the guest–host polymer films had the typical thicknesses of 3.0–3.5 μm, supporting 3–4 observable guided modes for high-accuracy determination of both refractive index and thickness.

From the multi-mode calculation, the standard deviations of the refractive-index measurements were typically 0.00005–0.0004 (or less than 0.03%), and those of thickness measurements were 3–10 nm (or less than 0.3%). For well-clamped samples, the recorded reflectivity showed minimal fluctuation across repeated measurements, typically around ±0.5%.

The accuracy of ATR-based EO measurements was fully verified by using benchmark EO materials, including thin-film lithium niobate (TFLN) and guest–host polymer SEO100.

### 2.2 Determination of the Order Parameter, *μ* and *α* for Chromophores from the Optical Birefringence of Poled Films

At first, the Sellmeier dispersion formula is used to fit the refractive index (*n*) of guest–host polymers by

1 $$n^{2}=n_{0}^{2}+\frac{A\lambda^{2}}{\lambda^{2}-\lambda_{0}^{2}}$$

where the first term (*n*_0_) represents the nonresonant constant corresponding to the static contribution from high-energy electronic absorption, the second term accounts for the primary resonance contribution from low-energy electronic absorption of the chromophore, *A* is a constant proportional to the oscillator strength, *λ* is the vacuum wavelength, and *λ*_0_ is the wavelength of maximum absorption.^22^ It should be noted that eq 1 follows the conventional form using *n* ^2^ and *n*_0_^2^, rather than *n* and *n*_0_ as proposed by Page et al. (Supplementary Note 1),^23^ for a more accurate fitting needed for the calculation of the order parameter.

Assuming that only dipole orientation effects of chromophores at low loading density are significant, and that both unpoled and poled guest–host polymers have the same index dispersion as described in eq 1, the order parameter (⟨*P*_2_⟩), which characterizes the first nontrivial axial order of the chromophore dipoles, can be determined from the ATR-measured refractive indices of poled films as follows

2 $$\left\langle P_{2}\right\rangle =\frac{3\left\langle \cos^{2}\theta \right\rangle -1}{2}=\frac{n_{\mathrm{TM}}-n_{\mathrm{TE}}}{n_{\mathrm{TM}}+2n_{\mathrm{TE}}-3n_{0}}$$

where *θ* is the angle between the chromophore dipole moment and the poling field. The order parameter ⟨*P*_2_⟩ is directly proportional to the poling-induced optical birefringence (Figure 2).^18,23^

**Figure 2:**
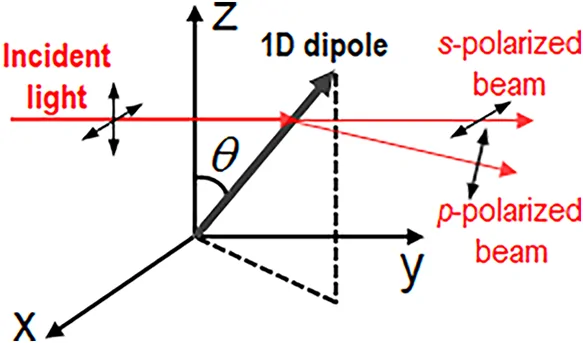
Orientation of a 1D chromophore dipole (thick arrow) in a (*x*, *y*, *z*) system. The dipole axis is tilted at the polar angle *θ* relative to the *z*-axis (poling field). Interaction with the dipoles in poled films can resolve an unpolarized incident light into *s*- and *p*-polarized components.

As the bulk material is azimuthally symmetric about the laboratory *z*-axis, Kuzyk derived that the bulk response of an ordered dilute ensemble of molecules has a first-order response as a function of ⟨*P*_2_⟩

3 $$\chi_{\mathrm{zz}}^{\left(1\right)}=\frac{N}{3}\left(1+2\left\langle P_{2}\right\rangle \right)\alpha_{33}^{*}$$

4 $$\chi_{\mathrm{xx}}^{\left(1\right)}=\chi_{\mathrm{yy}}^{\left(1\right)}=\frac{N}{3}\left(1-\left\langle P_{2}\right\rangle \right)\alpha_{33}^{*}$$

Here, *χ* ^(1)^ denotes the linear susceptibility, *N* is the chromophore loading density, and *α*_33_^*^ is the nonvanishing component of dressed polarizability of chromophore in the presence of the external field and host medium.^19^

Kuzyk further treated the guest–host polymer as a two-component system that includes both the polymer host and the dopant chromophore. The linear susceptibility *χ*_*ij*_^(1)^ of such a two-component system can be written as

5 $$\chi_{\mathrm{ij}}^{\left(1\right)}=\chi_{\mathrm{ij}}^{\left(1\right)\mathrm{poly}}+\chi_{\mathrm{ij}}^{\left(1\right)\mathrm{dye}}=\chi_{\mathrm{ij}}^{\left(1\right)\mathrm{poly}}+N<\alpha_{\mathrm{ij}}^{*}>$$

Here, the subscripts *i* and *j* refer to the coordinate system of the bulk material, *χ*_*ij*_^(1)*poly*^ is the polymer’s linear susceptibility, and *χ*_*ij*_^(1)*dye*^ is the dopant dye contribution as a function of *N*.^18^ The two independent diagonal components of *χ* ^(1)^ thus become

6 $$\chi_{\mathrm{zz}}^{\left(1\right)}=\chi_{\mathrm{zz}}^{\left(1\right)\mathrm{poly}}+\frac{N}{3}\left(1+2\left\langle P_{2}\right\rangle \right)\alpha_{33}^{*}$$

7 $$\chi_{\mathrm{xx}}^{\left(1\right)}=\chi_{\mathrm{yy}}^{\left(1\right)}=\chi_{\mathrm{xx}}^{\left(1\right)\mathrm{poly}}+\frac{N}{3}\left(1-\left\langle P_{2}\right\rangle \right)\alpha_{33}^{*}$$

Notably, the sum *χ*_*zz*_^(1)^ + *χ*_*xx*_^(1)^ + *χ*_*yy*_^(1)^ is conserved regardless of ⟨*P*_2_⟩, as expected from its dependence on *χ* ^(1)*poly*^, *N*, and *α*_33_^*^.^19,23^ Using the Gaussian (CGS) units, *χ* ^(1)^ values are derived from the refractive indices for guest–host polymers and *n*_0_^*poly*^ for the isotropic host polymer by

8 $$n_{\mathrm{TM}}={\left(1+4\pi \chi_{\mathrm{zz}}^{\left(1\right)}\right)}^{1/2}$$

9 $$n_{\mathrm{TE}}={\left(1+4\pi \chi_{\mathrm{xx}}^{\left(1\right)}\right)}^{1/2}$$

10 $$n_{0}^{\mathrm{poly}}={\left(1+4\pi \chi_{\mathrm{xx}}^{\left(1\right)\mathrm{poly}}\right)}^{1/2}$$

For the isotropic host polymer, *χ*_*zz*_^(1)*poly*^ = *χ*_*xx*_^(1)*poly*^. The contribution of *n*_0_^*poly*^ to the first-order response is considered approximately constant, independent of *N*, poling fields, and long-wavelength dispersion.^19^ It therefore has the same physical meaning as the *n*_0_ in eq 1, representing the nonresonant background contribution.

From eqs 6 and 7, the *α*_33_^*^ value of chromophore and ⟨*P*_2_⟩ can be determined as

11 $$\alpha_{33}^{*}={(\chi }_{\mathrm{zz}}^{\left(1\right)}+2\chi_{\mathrm{xx}}^{\left(1\right)}-3\chi_{\mathrm{xx}}^{\left(1\right)\mathrm{poly}})/N$$

12 $$\left\langle P_{2}\right\rangle =\frac{\chi_{\mathrm{zz}}^{\left(1\right)}-\chi_{\mathrm{xx}}^{\left(1\right)}}{\chi_{\mathrm{zz}}^{\left(1\right)}+2\chi_{\mathrm{xx}}^{\left(1\right)}-3\chi_{\mathrm{xx}}^{\left(1\right)\mathrm{poly}}}≅\frac{n_{\mathrm{TM}}-n_{\mathrm{TE}}}{n_{\mathrm{TM}}+2n_{\mathrm{TE}}-3n_{0}}$$

As the poling efficiency depends on the electrostatic dipole alignment energy relative to thermal energy, the order parameter ⟨*P*_2_⟩ can also be written as a function of *u* = μ*E_p_/(*kT*)­

13 $$\left\langle P_{2}\right\rangle =1+\frac{3}{u^{2}}-\frac{3}{u}\coth(u)$$

where the μ* is the dressed dipole moment that couples to the external field to produce a torque, *E*_p_ is the applied poling field, *k* is Boltzmann’s constant, and *T* is the poling temperature in Kelvin.^18,19^ Therefore, the μ* of dipolar chromophore can be determined from the measured ⟨*P*_2_⟩ that can derive the *u* (Figure S1).

In the low-field limit, the ⟨*P*_2_⟩ and poling-induced optical birefringence can be approximated as

14 $$\left\langle P_{2}\right\rangle \approx \frac{1}{15}u^{2}$$

15 $$n_{\mathrm{TM}}-n_{\mathrm{TE}}=\frac{2\pi N}{n_{0}}\left\langle P_{2}\right\rangle \alpha_{33}^{*}=\frac{2\pi N}{15n_{0}}u^{2}\alpha_{33}^{*}$$

This approximation remains valid, provided that *u* ≤ 1 and the short-range interaction potential *U*_int_ between the molecules is negligible.^18,19,23^

### 2.3 Determination of *r*-Coefficients for the Poled Films from DC- and AC-Modulated ATR Measurements

The DC *r*-coefficients of a poled film with thickness *d* were determined by measuring the refractive-index change induced by an applied DC modulation voltage *V*_*mod*_^DC^ via the Pockels’ effect^10,11,13,15^

16 $$\Delta \left(\frac{1}{n_{\mathrm{TE}}^{2}}\right)=r_{13}^{\mathrm{DC}}V_{\mathrm{mod}}^{\mathrm{DC}}/d$$

17 $$\Delta \left(\frac{1}{n_{\mathrm{TM}}^{2}}\right)=r_{33}^{\mathrm{DC}}V_{\mathrm{mod}}^{\mathrm{DC}}/d$$

Based on the same principle, the AC *r*-coefficients of the poled film were determined by

18 $$r_{13}=-\frac{2dN_{\mathrm{eff}}}{n_{\mathrm{TE}}^{4}V_{\mathrm{mod}}}\Delta R_{\mathrm{TE}}/\left(\frac{\partial R_{\mathrm{TE}}}{\partial N_{\mathrm{eff}}^{s}}\right)$$

19 $$r_{33}=-\frac{2d}{n_{\mathrm{TM}}^{2}N_{\mathrm{eff}}V_{\mathrm{mod}}}\Delta R_{\mathrm{TM}}/\left(\frac{\partial R_{\mathrm{TM}}}{\partial N_{\mathrm{eff}}^{p}}\right)$$

where *V*_*mod*_ is the modulation voltage, *R* represents the measured reflectivity as a function of the effective refractive index (*N*_eff_) in ATR spectra, Δ*R* denotes the modulated reflectivity change, and *N*_eff_^*s*^ and *N*_eff_^*p*^ are the effective refractive indices for *s*- (or TE) and for *p*-polarized (TM) waves, respectively. The ∂*R*/∂*N* derivatives are inherent in the ATR spectra.^24–28^ The negative sign in eqs 18 and 19 is sometimes omitted.

### 2.4 Estimation of *β*_μ_ Values for Chromophores from *r*-Coefficients

According to ROGM, the noncentrosymmetric order parameter ⟨cos^3^ *θ*⟩ or the third Langevin function *L*_3_(*u*) for poled films is related to the dipole alignment energy *u* = μ*E_p_/(*kT*) by

20 $$\left\langle \cos^{3}\theta \right\rangle \equiv L_{3}\left(u\right)=\left(1+\frac{6}{u^{2}}\right)\coth(u)-\frac{3}{u}\left(1+\frac{2}{u^{2}}\right)$$

The *r*_33_ value of poled films (in mks units) is then expressed as

21 $$r_{33}=2N\beta_{\mu }^{*}\left(-\omega ;\omega ,0\right)\left\langle \cos^{3}\theta \right\rangle /n_{\mathrm{TM}}^{4}$$

where *β*_μ_^*^(−ω; ω, 0) denotes the dressed vector component of *β* tensors incorporating the local field factors along the direction of the chromophore dipole moment, and ω represents the optical frequency.^16,18^ With the measured *r*_33_, *N*, *n*_TM_, and derived *u* from ⟨*P*_2_⟩, the *β*_μ_^*^(−ω; ω, 0) can be calculated using eqs 20 and 21.

This framework represents a classic thermodynamic model for the quantitative analysis of guest–host EO polymers, without requiring extensive computational and experimental effort.^16–19^ It provides a practical means of directly estimating *β*_μ_^*^(−ω; ω, 0) values of chromophores in structure–property relationship studies of poled polymers at the wavelength relevant to EO waveguides.^29^ Unlike second-harmonic-based techniques, this approach avoids the associated experimental and analytical complexities while complementing methods such as electric-field-induced second-harmonic generation (EFISH) and hyper–Rayleigh scattering (HRS) for measuring molecular *β*(−2ω; ω, ω) values of chromophores in solution.^10,11,13,15,29^

## 3 Results and Discussion

### 3.1 Study of ⟨*P*_2_⟩, *μ*, and *α* of a Benchmark PPH-Phore M1a-FP-PhON in Poled Guest–Host Polymer from the First-Order Responses

We started our analysis using a benchmark PPH-phore, **M1a-FP-PhON** (Chart 1). It features modular, scalable synthesis, excellent chemical and thermal stability, and large hyperpolarizability.^13,15^ A guest–host polymer was prepared by doping 21.43 wt % of **M1a-FP-PhON**, corresponding to a number density *N* of 1.30 × 10^20^ cm^–3^, into the host P­(S-*co*-MMA). The films of this polymer, denoted as **M1a-FP-PhON**/P­(S-*co*-MMA)/*N*1.30, were poled at different field strengths from 21 to 103 V/μm, with the maximum poling temperature of 115 °C.^30^

**Chart 1:**
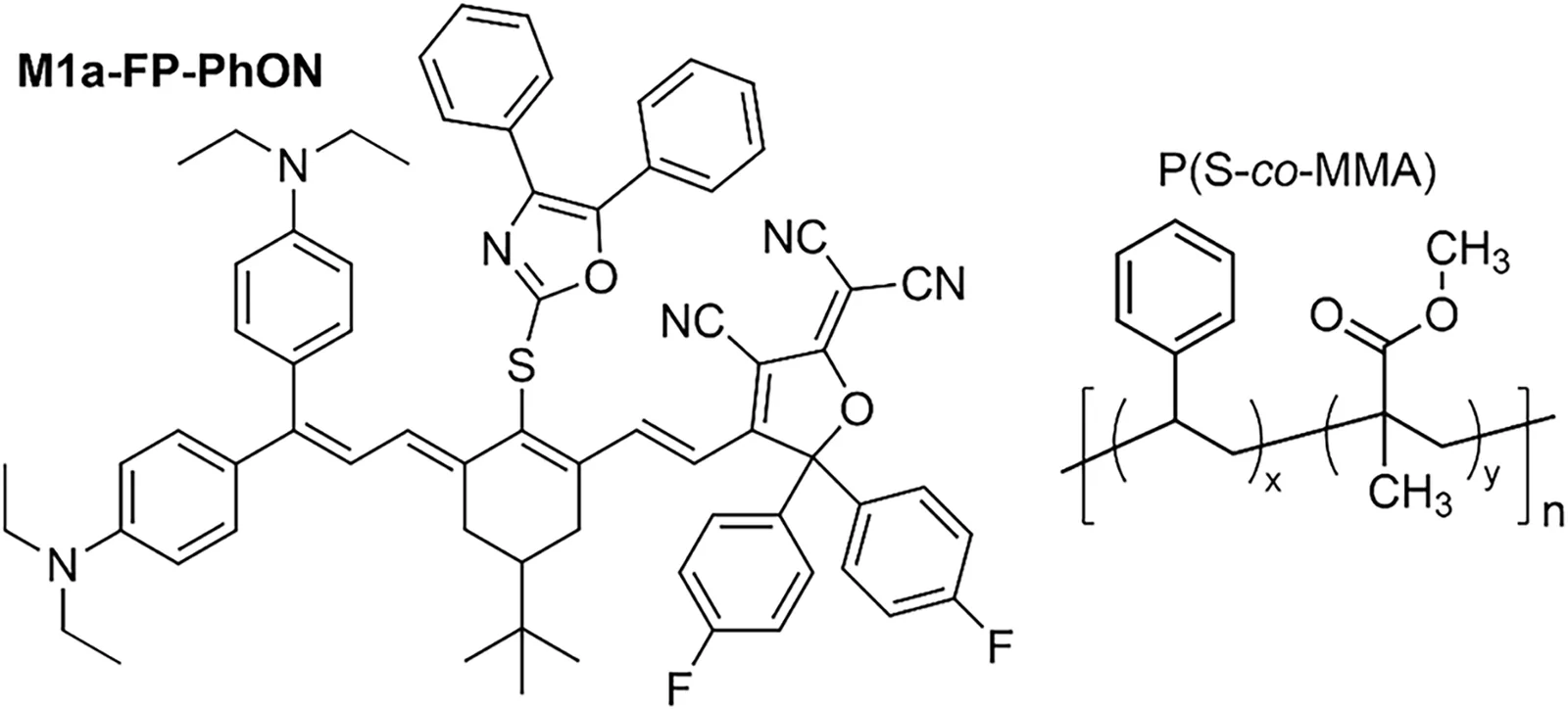
Benchmark PPH-Phore M1a-FP-PhON and Host Polymer P­(S-*co*-MMA) for This Study

The ATR spectra of both unpoled and poled films (Figure 3a–d) clearly reveal the transition from an isotropic to a uniaxially anisotropic state. In the unpoled film, the left-most TE and TM modes are very close, indicating nearly identical *n*_TE_ and *n*_TM_. Fitting the refractive indices of unpoled films at 1304 and 1541 nm gives the Sellmeier dispersion formula of **M1a-FP-PhON**/P­(S-*co*-MMA)/*N*1.30 by

22 $$n^{2}=1.49585^{2}+\frac{0.2307\lambda^{2}}{\lambda^{2}-862^{2}}$$

**Figure 3:**
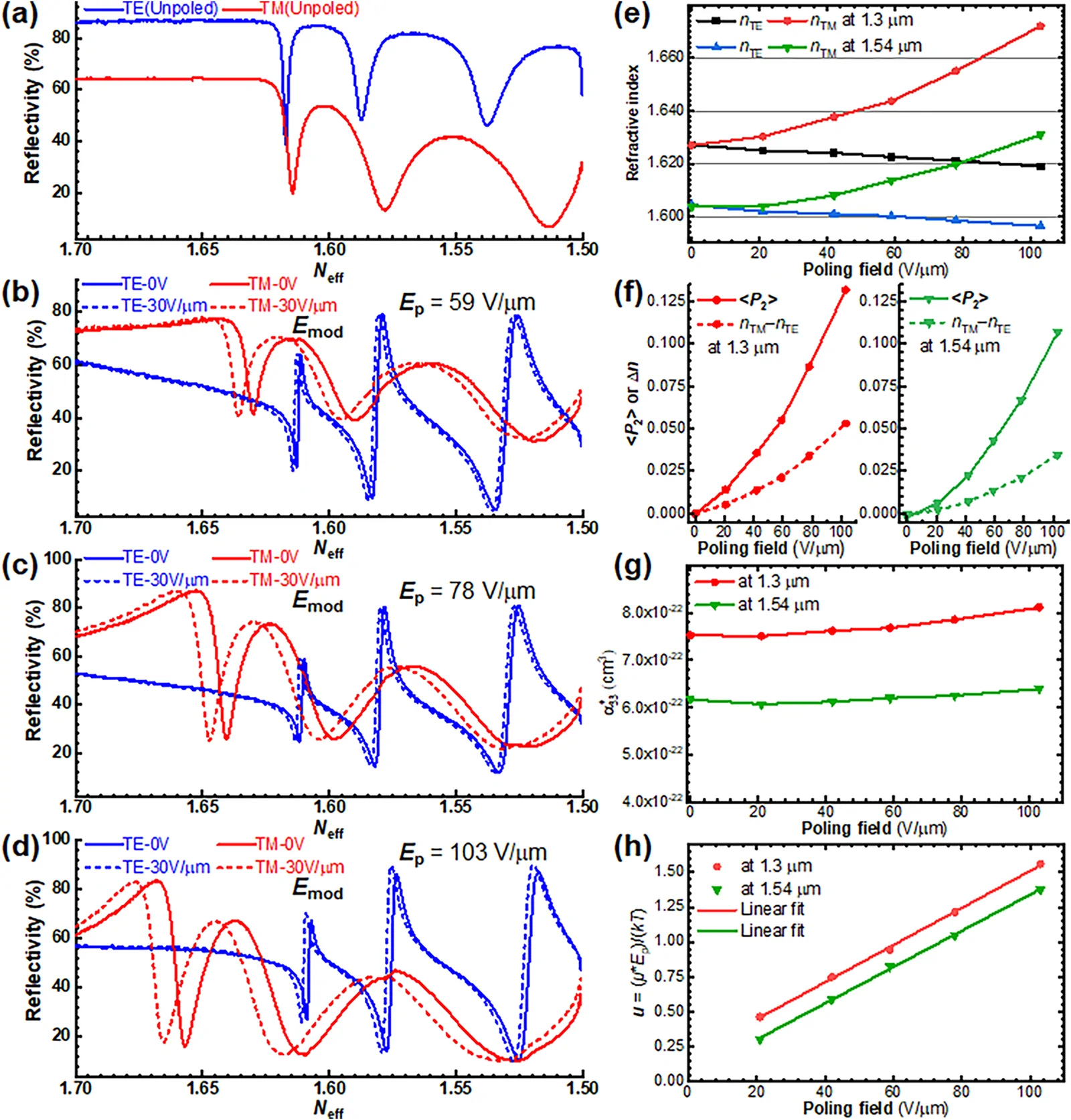
ATR characterization and analysis of poling-induced optical anisotropy in **M1a-FP-PhON**/P­(S-*co*-MMA)/*N*1.30 with film thickness of 3.1–3.2 μm. (a–d) ATR spectra of reflectivity versus effective refractive index (*N*_eff_) for TE (blue) and TM (red) modes at unpoled and various poled states. Solid lines represent the unpoled and poled films under zero bias (0 V), and dashed lines indicate the EO-induced shift in guided modes under a positive DC modulation field (*E*_*mod*_) of 30 V/μm. (e) Evolution of refractive indices at 1.3 and 1.54 μm as a function of *E*_p_. (f) Calculated ⟨*P*_2_⟩ and measured optical birefringence vs *E*_p_. (g) Poling-field-dependent *α*_33_^*^ for both operation wavelengths. (h) Linear relationship between the alignment energy parameter *u* and *E*_p_, with solid lines representing linear fits to the experimental data.

Here, the first term *n*_0_ of 1.49585 is close to the index of P­(S-*co*-MMA), and 862 nm in the second term is the maximum absorption wavelength of the films.

For poled films, as the poling field *E*_p_ increases, the TE and TM modes progressively diverge. Specifically, the TM modes shift significantly toward a higher effective refractive index (*N*_eff_), whereas the TE modes shift slightly toward lower values. This indicates that the dipolar chromophores, initially randomly oriented, are being driven to align along the poling field direction. As a result, the refractive index along the film normal (*n*_TM_) increases, while the in-plane components (*n*_TE_) decrease.

Quantitatively, this poling-induced anisotropy is captured in Figure 3e-f, where the birefringence (Δ*n* = *n*_TM_ – *n*_TE_) widens substantially as the field strength reaches 103 V/μm. The dispersion between 1.3 and 1.54 μm is consistent with the electronic resonance properties of the chromophore: the higher refractive indices and more pronounced shifts at 1.3 μm are due to its closer proximity to the material’s absorption band.

The thermodynamic nature of poling-induced alignment is validated in Figure 3f–h. Both the calculated ⟨*P*_2_⟩ from eq 12 and the experimental birefringence Δ*n* exhibit near-quadratic dependence on *E*_p_, consistent with the low-field analysis described by eqs 14 and 15.^18,19,23^ The standard deviation for ⟨*P*_2_⟩ values measured at multiple spots within the poled area is approximately 0.0002–0.0005 for efficiently poled films at field strengths above 60 V/μm, compared with 0.001–0.002 for weakly poled films under low fields of 20–40 V/μm. These results demonstrate the high accuracy of ⟨*P*_2_⟩ analysis, which can be attributed to the excellent absolute accuracy and resolution of the refractive-index measurement using the Metricon 2010/M.

Using eq 11, the *α*_33_^*^ values of **M1a-FP-PhON** are calculated to be ∼7.5 × 10^–22^ cm^3^ at 1.3 μm and ∼6.1 × 10^–22^ cm^3^ at 1.54 μm, consistent with the refractive-index dispersion presented in Figure 3e. Because these values are determined solely from the refractive indices of unpoled and poled films, they inherit the same high level of accuracy, with a standard deviation below 0.03%. The slight increase in *α*_33_^*^ versus *E*_p_ is attributed to a subtle field-induced enhancement of the electronic response and Lorentz–Lorenz local field factor, as dipoles align more efficiently.

The dimensionless alignment energy *u* = μ*E_p_/(*kT*), obtained from eq 13, shows a highly linear relationship with *E*_p_ (Figure 3h). Notably, the slopes at both wavelengths are identical, quantifying the μ* of 20.9 debye for **M1a-FP-PhON**, independent of the probe optical frequency. The excellent linear fit (*R* ^2^ > 0.99) and small relative standard error of 1.6% indicate that chromophore orientation in guest–host polymers follows the ROGM prediction of a freely rotating dipole in a viscous medium during poling. This excellent linearity further supports the assumption that mean-field intermolecular interactions are isotropic and that μ* remains constant across the experimental range as the primary molecular property governing polar order.^16–19^

Interestingly, the linear fit in Figure 3h displays a nonzero intercept at 1.3 μm, whereas the fit at 1.54 μm lies only slightly above zero. This offset is attributed to increased optical absorption at 1.3 μm, which is closer to the chromophore’s absorption edge, compared to the more transparent regime at 1.54 μm.

### 3.2 Study of *r*_33_ and *β*_μ_ Values of M1a-FP-PhON in Poled Guest–Host Polymers from DC- and AC-Modulated ATR Spectroscopy

For poled guest–host polymers, DC-modulated ATR measurements are straightforward, as the *r*-coefficients can be directly determined from the field-induced mode shift under applied fields from 10 to 30 V/μm.^13^ This simplicity makes DC measurements a practical approach for rapidly estimating the EO activities of poled polymers. The obtained DC *r*-coefficients are also directly relevant to device design, as they quantify the bias voltages required to control the operating points of waveguide modulators.^31–33^ Moreover, DC measurements serve as a valuable reference for interpreting AC-modulated EO measurements, including the determination of sign and asymmetric EO responses in poled films.^34^

Under the positive DC modulation voltages, the **M1a-FP-PhON**/P­(S-*co*-MMA)/*N*1.30 film poled at a high field of 103 V/μm exhibited a large *r*_33_ value of −124.7 pm/V at 1.3 μm and −69.3 pm/V at 1.54 μm. The negative sign denotes an increase in refractive indices under the positive DC modulation voltages (Figure 3b-d). Under the negative DC voltages, however, the measured *r*_33_ values for the same poled films were 20% to 25% lower than those obtained under the positive voltages (Figure S2). This is not unexpected for DC measurements, where the effective field strengths under reversed polarity are reduced due to screening by homocharges accumulated at the film interfaces.^34^

As shown in Figure 4a, the *r*_33_ values exhibit a dependence on the poling field that essentially follows the third-order Legendre polynomial.^16,18^ Notably, the wavelength dispersion of the EO responses is more pronounced than that of the first-order optical properties.

**Figure 4:**
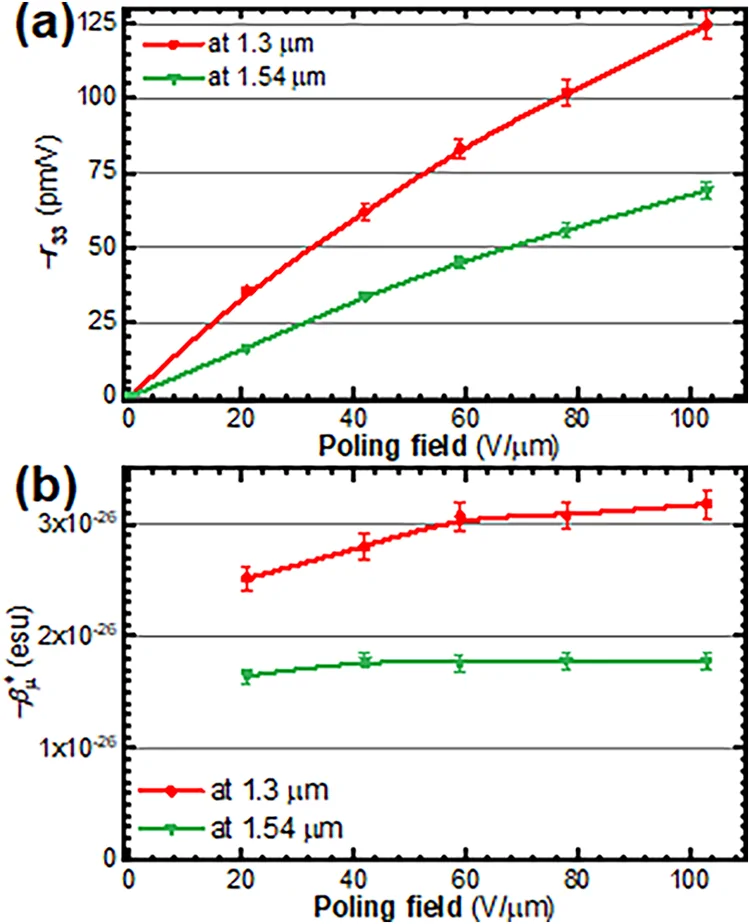
EO coefficient and hyperpolarizability analysis of **M1a-FP-PhON**/P­(S-*co*-MMA)/*N*1.30 from ATR measurements under the positive DC modulation voltages. (a) DC *r*_33_ values at 1.3 and 1.54 μm as a function of poling field *E*_p_. (b) Microscopic molecular *β*_μ_^*^ values calculated via ROGM across the poling-field range. The error bar is the relative standard deviation (RSD) of 4.0% from the experiments (worst cases).

Using the ROGM transformation of eqs 20 and 21, the *β*_μ_^*^ values were plotted against the poling field in Figure 4b. Remarkably, the extracted dressed *β*_μ_^*^ values of **M1a-FP-PhON** reach approximately −31,000 × 10^–30^ esu at 1.3 μm and −18,000 × 10^–30^ esu, representing ultralarge molecular nonlinearities that rank among the highest values ever reported for poled polymer systems. These values remain nearly constant across modest to high poling fields, while slightly reduced values obtained at 21 V/μm may arise from the residual centrosymmetric chromophore packing in weakly poled films. Overall, the results confirm the excellent applicability of ROGM to the guest–host system **M1a-FP-PhON**/P­(S-*co*-MMA)/*N*1.30.

Next, we conducted modulated ATR characterization under AC voltages to quantify the EO response of poled films under periodically varying electric fields.^27,28^ This approach enables a more precise analysis of the dynamic and frequency-dependent EO response. The method is streamlined through direct ATR detection using the Metricon 2010/M prism coupler, eliminating the need for a lock-in amplifier or oscilloscope, and has been validated with commercial TFLN and benchmark EO polymers. Systematic ATR measurements as a function of waveform, applied voltage, and modulation frequency quantitatively isolate the pure electronic response of EO films in a prism-coupled slab waveguide configuration while ruling out contributions from mechanical and thermal effects.^27,28^

As shown in Figures 5a,5d, applying a 20 V square-wave modulation at 6 Hz induces numerous measurable oscillations in the guided modes. The direct subtraction of the modulated states yields the differential reflectivity spectrum (Δ*R*, red curves), which exhibits sharp bipolar features maximized at the highest slopes (first derivatives) of the resonance dips.

**Figure 5:**
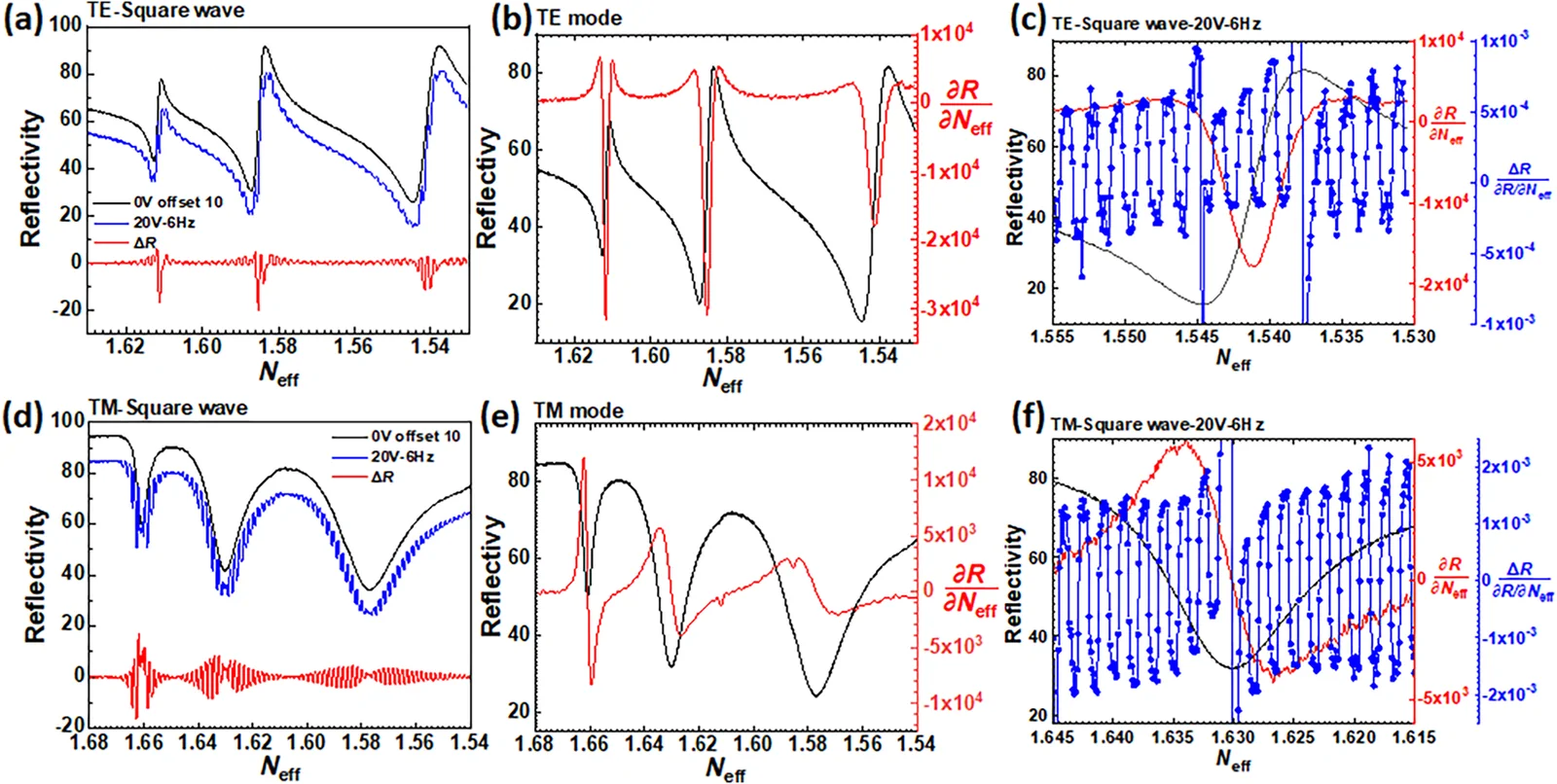
Lock-in-free ATR characterization of a poled **M1a-FP-PhON**/P­(S-*co*-MMA)/*N*1.30 film by the poling field 100 V/μm with film thickness of 3.52 μm. (a) TE-mode reflectivity spectra under 0 V (unmodulated, black curve) and under a 20 V, 6 Hz square-wave voltage (blue curve), as well as the extracted spectrum of differential reflectivity (Δ*R*, red curve). (b) Measured TE-mode reflectivity spectrum plotted with the calculated analytical first derivative (red curve). (c) High-resolution view of the raw modulated TE reflectivity signal by a 20 V, 6 Hz square-wave voltage, directly capturing the modulated effective index change (blue curve) along with the reflectivity spectrum and its analytical first derivative (red curve). (d–f) Corresponding TM-mode data sets matching the structure of the TE-mode profiles in panels (a–c).

To confirm that these variations originate primarily from voltage-induced index modulation, the experimental reflectivity spectra were compared directly to its first derivative with respect to *N*_eff_ in Figures 5b and 5e. The excellent line-shape alignment between the experimental Δ*R* peaks and the analytical derivative extrema verifies that the signal follows the first-order EO response of the poled films, as described by eqs 18 and 19.

The EO-induced index changes captured under the AC modulation voltages are highlighted in Figures 5c and 5f. The dense vertical oscillations represent the real-time switching of the film’s refractive-index change between the positive and negative voltages under square modulation waves, amplified by the substantial Δ*R* at the steepest regions of the ATR spectra.

Table 1 summarizes the refractive indices and *r*-coefficients for the poled **M1a-FP-PhON**/P­(S-*co*-MMA)/*N*1.30 film by modulated ATR spectroscopy. The measured Pockels coefficients are highly consistent under different modulation conditions (DC, sinusoidal wave, and square wave).

**Table 1:** Summary of the Refractive Indices and EO Coefficients for a Poled **M1a-FP-PhON**/P­(S-*co*-MMA)/*N*1.30 Film by Modulated ATR Spectroscopy

wavelength	*n*_TE_/*n*_TM_	Pockels coefficient	DC	sine wave 6 Hz	square wave 6 Hz
1.3 μm	1.61940/1.66950	*r*_13_ (pm/V)	–33.5	–30.6	–32.1
		*r*_33_ (pm/V)	–115.5	–117.2	–119.5
1.54 μm	1.59839/1.63063	*r*_13_ (pm/V)	–26.1	–22.5	–26.4
		*r*_33_ (pm/V)	–67.1	–70.7	–72.9

### 3.3 Study of Loading-Density-Dependent Properties of M1a-FP-PhON in Poled Guest–Host Polymers from Modulated ATR Spectroscopy

To evaluate the effect of chromophore loading on the molecular and bulk properties, the refractive indices of both unpoled and poled guest–host polymer films were measured as a function of *N*, ranging from 0.7 × 10^20^ to 1.7 × 10^20^ cm^–3^. As shown in Figure 6a and Table S1, the refractive indices of the unpoled films increase linearly with *N*. From Sellmeier fitting, the *n*_0_ of the background index remains nearly constant at ∼1.50, while the dispersion coefficient *A* increases linearly with *N*. Upon poling under an applied field of ∼100 V/μm, a significant birefringence was observed across all loading densities. In a relatively dilute regime (*N* ≤ 1.3 × 10^20^ cm^–3^), the DC *r*_33_ values exhibit a linear dependence on the number density. At an optimal *N* of 1.3 × 10^20^ cm^–3^, the poled film achieved peak DC *r*_33_ values of −125 pm/V at 1.3 μm and −90 pm/V at 1.54 μm, which differ slightly from the values in Table 1 due to the use of different poling conditions. Further increasing *N* triggers a sharp roll-off in *r*_33_, indicating strong electrostatic interactions and enhanced conductivity at high loading severely limit the achievable DC-modulated EO activity of materials.^15,34^

**Figure 6:**
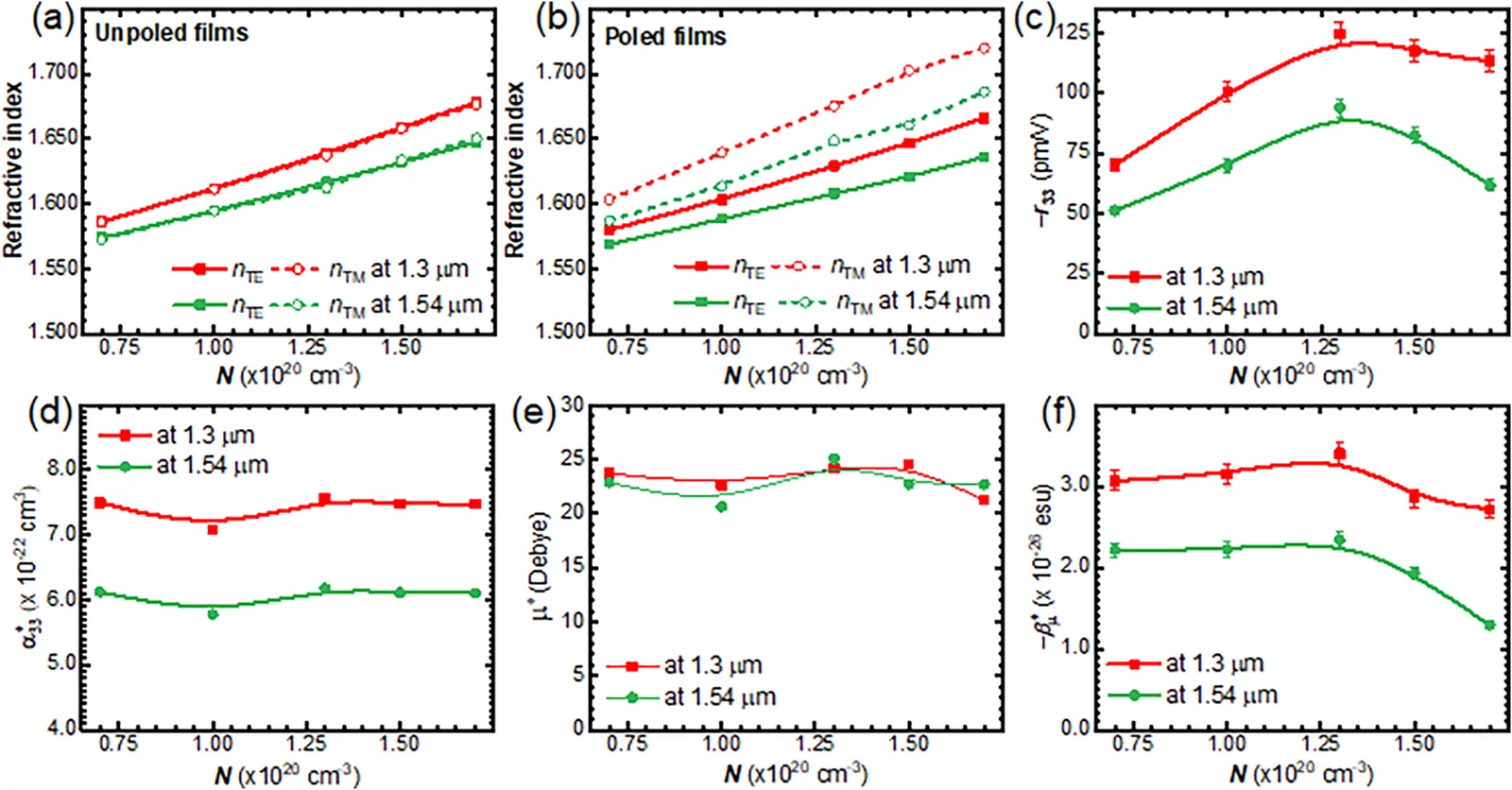
Loading-density-dependent optical, EO, and effective molecular properties of **M1a-FP-PhON**/P­(S-*co*-MMA) films at 1.3 and 1.54 μm. Refractive indices of (a) unpoled and (b) poled films versus *N*. (b) *r*_33_ values as a function of *N*. (c) *α*_33_^*^, (d) μ*, and (e) *β*_μ_^*^ values evaluated at the different loading densities. The error bar in panels (b) and (e) is the RSD of 4.0% from the experiments (worst cases). The experimental and analytical data points are connected through the B-spline curves.

Notably, the *α*_33_^*^ values of **M1a-FP-PhON** remain essentially constant at ∼7.5 × 10^–22^ cm^3^ at 1.3 μm and ∼6.1 × 10^–22^ cm^3^ at 1.54 μm. The results are highly consistent with the poling-field-dependent study presented in Figure 3g. Concurrently, the μ* from the single-*E*_p_ calculation is estimated to be around 22–24 D, which matches the field-dependent control results. More importantly, the calculated ultralarge *β*_μ_^*^ values of **M1a-FP-PhON** remain highly stable up to the optimal *N* threshold. The results show that ROGM is applicable over the *N* range of 0.7 × 10^20^ to 1.3 × 10^20^ cm^–3^, where the short-range interaction potential between chromophores is expected to be very small. As the μ* results incorporate the Onsager local field factor, the experimentally determined dipole moment of **M1a-FP-PhON** is substantially lower than the values calculated by density functional theory (DFT), up to 25 D in gas phase and 37 D in chloroform.^13^ This comparison further indicates that the intermolecular interactions between **M1a-FP-PhON** are negligible under the conditions relevant to the ROGM analysis, particularly during poling at elevated temperatures.

Accordingly, the ROGM captures the dominant structure–property relationships under the experimental conditions. The decline in performance at higher *N*s is therefore attributed primarily to reduced poling efficiency rather than a degradation of intrinsic molecule parameters. As we have recently reported, the problem can be alleviated by introducing an asymmetrical donor to modify the structure of **M1a-FP-PhON**, resulting in an enhanced EO coefficient of 149.1 pm/V at 1.3 μm.^15^

To evaluate the theoretical potential of **M1a-FP-PhON**/P­(S-*co*-MMA) guest–host systems, we plotted the dependence of refractive indices and *r*_33_ values on *N* using the two-component analysis and ROGM by eqs 6–10, 20 and 21 (Figure 7).^18,19^ Assuming that the intrinsic ⟨*P*_2_⟩ of **M1a-FP-PhON** can be achieved at high *N* through molecular engineering, ROGM predicts a highly linear increase in both *n*_TE_ and *n*_TM_ across a broad loading range up to 5.0 × 10^20^ cm^–3^, which corresponds to an ultrahigh 82.4 wt % of **M1a-FP-PhON** in the guest–host formulation. The calculated large birefringence reflects a high degree of polar order under efficient poling conditions. Utilizing the molecular parameters of **M1a-FP-PhON** determined in this study, the predicted *r*_33_ values climb steeply at lower loading densities, as demonstrated in our experiments. At a maximum *N* of 5.0 × 10^20^ cm^–3^, the **M1a-FP-PhON**/P­(S-*co*-MMA) guest–host system is projected to reach an exceptional theoretical *r*_33_ of −198.3 pm/V (*n*_TM_ = 2.050) at 1.3 μm, and −133.1 pm/V (*n*_TM_ = 1.953) at 1.54 μm. This simulation highlights the unexploited potential and room for performance optimization of **M1a-FP-PhON**.

**Figure 7:**
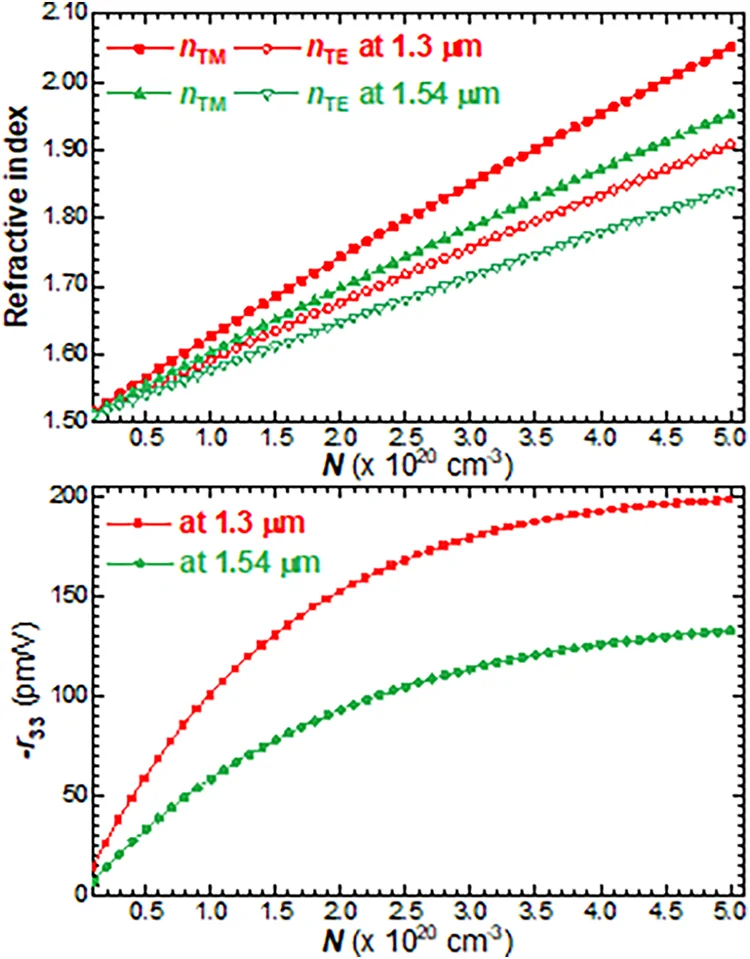
Dependence of (top) refractive indices and (bottom) *r*_33_ values on *N*, analyzed via the two-component analysis and ROGM based on the following parameters for **M1a-FP-PhON**/P­(S-*co*-MMA) films: *α*_33_^*^ of 7.5 × 10^–22^ cm^3^, ⟨*P*_2_⟩ of 0.120, and *β*_μ_^*^ of –31,000 × 10^–30^ at 1.3 μm; *α*_33_^*^ of 6.1 × 10^–22^ cm^3^, ⟨*P*_2_⟩ of 0.110, and *β*_μ_^*^ of –18,000 × 10^–30^ at 1.54 μm.

### 3.4 Quantifying the Molecular and Bulk Responses in Guest–Host Systems Containing a Series of PPH-Phores in P­(S-*co*-MMA)

The analytical protocol is further applied to evaluate their molecular and bulk properties for guest–host systems containing a series of PPH-phores in P­(S-*co*-MMA), which have been reported previously with preliminary ATR characterization (Chart 2).^10,11,13,35^

**Chart 2:**
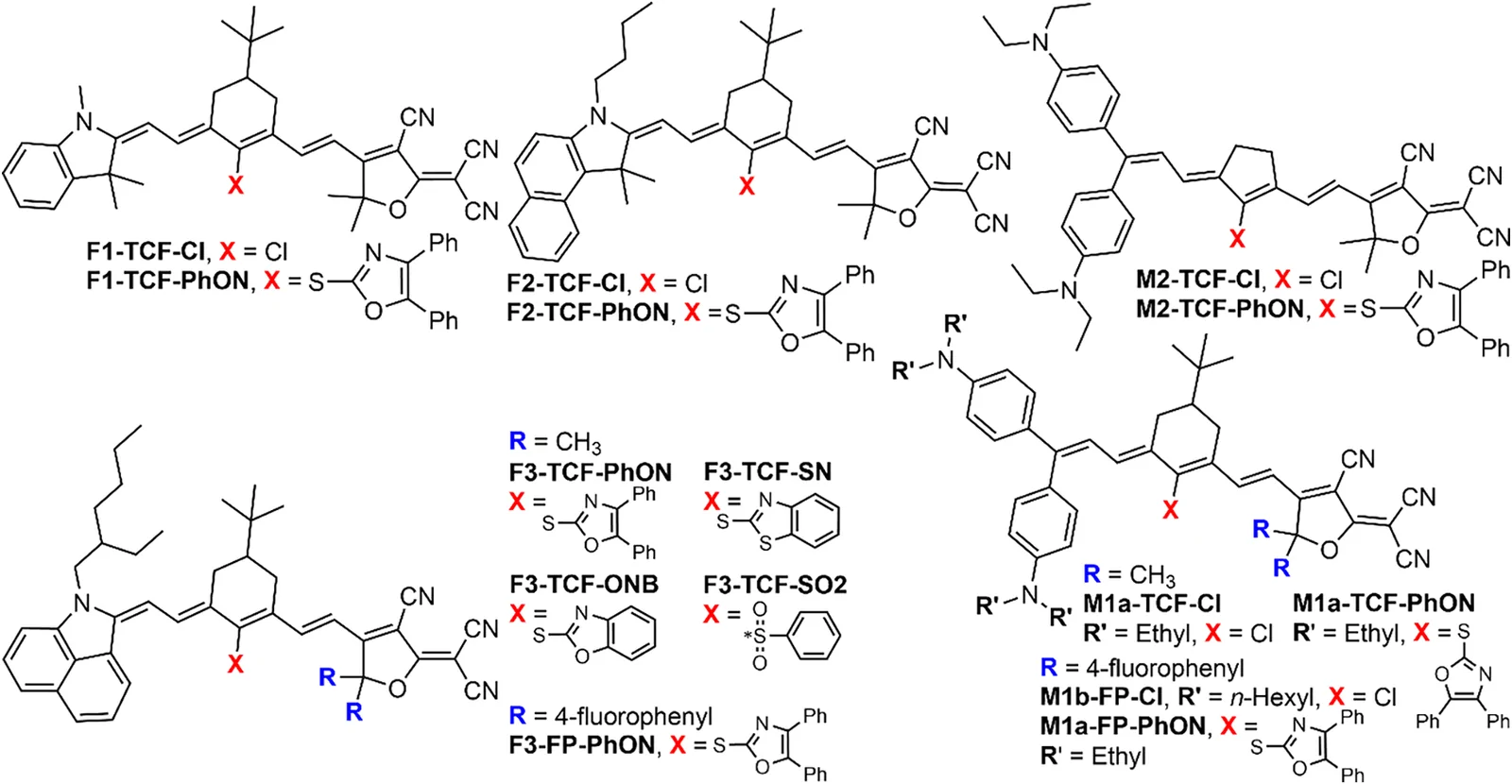
PPH-phores Containing Various Donors, *meso*-Substituents, and TCF Acceptors for Guest–Host EO Polymers[Fn c2fn1]

a The PPH-phores are named after the unique moieties in the sequence of donor–acceptor-meso-substituent.

The ATR-measured refractive indices of the poled guest–host films show distinct TE–TM index splitting at both 1.3 and 1.54 μm, consistent with poling-induced dipole alignment of the PPH-phores in poled films (Table 2). For highly efficient chromophores such as **M1a-FP-PhON** and **F3-FP-PhON**, the poling-induced increase in birefringence is particularly pronounced, reflecting their substantial contribution to the bulk susceptibility of poled films.^13^

**Table 2:** Summary of Optical and EO Properties of Guest–Host Polymers Containing a Series of PPH-Phores in P­(S-*co*-MMA) with *N* of 1.30 × 10^20^ cm^–3^

**PPH-phores** in P(S-*co*-MMA)	λ_max_ (nm)	unpoled films		poled films		–*r*_33_ (pm/V), DC^a^	
		*n*_TE_, *n*_TM_ at 1.3 μm	*n*_TE_, *n*_TM_ at 1.54 μm	*n*_TE_, *n*_TM_ at 1.3 μm	*n*_TE_, *n*_TM_ at 1.54 μm	1.3 μm	1.54 μm
**F1-TCF-Cl**	835	1.58884, 1.58966	1.57680, 1.57657	1.58406, 1.62329	1.57016, 1.60219	18.6	
**F1-TCF-PhON**	840	1.59826, 1.59690	1.58528, 1.58448	1.58988, 1.63653	1.57868, 1.61987	35.7	
**F2-TCF-Cl**	863	1.58721, 1.58839	1.57512, 1.57362	1.57480, 1.61837	1.56445, 1.59052	24.9	
**F2-TCF-PhON**	870	1.61292, 1.61195	1.59662, 1.59640	1.60159, 1.65169	1.58725, 1.63564	28.9	
**M2-TCF-Cl**	768	1.59232, 1.59112	1.57929, 1.57843	1.58394, 1.61258	1.57249, 1.59639	39.5	23.2
**M2-TCF-PhON**	791	1.58234, 1.58256	1.57102, 1.57046	1.57503, 1.60090	1.56448, 1.58494	55.5	39.2
**F3-TCF-PhON**	803	1.60784, 1.60698	1.59026, 1.58988	1.59559, 1.63529	1.58109, 1.61857	59.0	39.0
**F3-TCF-SN**	810	1.61116, 1.60989	1.59388, 1.59147	1.60216, 1.62925	1.58594, 1.60950	55.9	41.0
**F3-TCF-ONB**	806	1.60554, 1.60492	1.58886, 1.58816	1.59719, 1.62523	1.58223, 1.60378	55.8	
**F3-TCF-SO2**	811	1.59678, 1.59658	1.58324, 1.58189	1.58814, 1.61731	1.57576, 1.59967	54.1	
**F3-FP-PhON**	896	1.65762, 1.65557	1.62778, 1.62417	1.64720, 1.68582	1.62001, 1.65374	74.2	
**M1a-TCF-Cl**	743	1.58424, 1.58370	1.57371, 1.57368	1.57816, 1.60293	1.56860, 1.58951	47.4	38.7
**M1a-TCF-PhON**	768	1.59307, 1.59200	1.58145, 1.58087	1.58473, 1.61688	1.57456, 1.60153	63.4	48.3
**M1b-FP-Cl**	838	1.62297, 1.62168	1.60305, 1.60430	1.61332, 1.65352	1.59517, 1.62625	74.6	
**M1a-FP-PhON**	866	1.63885, 1.63724	1.61658, 1.61225	1.62925, 1.67514	1.60790, 1.64877	124.6	94.0

a The relative standard deviation (RSD) of these results varies from 3.2 to 7.0%, as previously reported.^11,35^

Using these ATR data, the two-component analysis through ROGM quantifies the order parameter ⟨*P*_2_⟩ and systematically evaluates the *α*_33_^*^, μ*, and *β*_μ_^*^ values of PPH-phores (Table 3). Overall, the analysis demonstrates the self-consistency of the ATR–ROGM framework in extracting intrinsic molecular parameters from macroscopic optical measurements.

**Table 3:** Derived ⟨*P*_2_⟩, *α*_33_^*^, μ*, and *β*_μ_^*^ Values for PPH-Phores from ATR Measurements of Guest–Host Polymers Shown in Table 2

**PPH-phores** in P(S-*co*-MMA) with *N* of 1.30 × 10^20^ cm^–3^	Sellmeier fitting constants		⟨*P*_2_⟩		*α*_33_* (10^–22^ cm^3^)		μ* (Debye)	–*β*_μ_* (10^–30^ esu)	
	*A*	*n*_0_	1.3 μm	1.54 μm	1.3 μm	1.54 μm	1.54 μm	1.3 μm	1.54 μm
**F1-TCF-Cl**	0.14041	1.51238	0.1589	0.1598	4.89	3.93	27.9	3820	
**F1-TCF-PhON**	0.14211	1.51989	0.1873	0.1943	4.96	4.19	31.8	6970	
**F2-TCF-Cl**	0.12735	1.51456	0.1995	0.1514	4.31	3.35	26.9	4510	
**F2-TCF-PhON**	0.15405	1.52409	0.1832	0.2092	5.49	4.60	33.4	5930	
**M2-TCF-Cl**	0.20415	1.49052	0.0960	0.0914	5.88	5.12	19.9	10,200	5760
**M2-TCF-PhON**	0.16269	1.49887	0.1047	0.0965	4.84	4.12	20.5	13,300	9050
**F3-TCF-PhON**	0.23396	1.48572	0.1121	0.1202	7.06	6.16	23.3	14,900	9450
**F3-TCF-SN**	0.22954	1.49021	0.0777	0.0785	6.95	5.92	18.2	16,800	11,700
**F3-TCF-ONB**	0.22082	1.48989	0.0833	0.0746	6.69	5.68	17.7	14,900	12,600
**F3-TCF-SO2**	0.17871	1.50268	0.1055	0.1011	5.47	4.64	21.1	13,500	
**F3-FP-PhON**	0.25983	1.50107	0.0853	0.0902	9.31	7.56	19.7	24,300	
**M1a-TCF-Cl**	0.18395	1.49561	0.0938	0.0896	5.19	4.55	19.7	12,100	9550
**M1a-TCF-PhON**	0.18124	1.50307	0.1198	0.1149	5.31	4.60	22.7	14,800	10,900
**M1b-FP-Cl**	0.21693	1.50435	0.1141	0.1060	7.10	5.83	21.7	19,500	
**M1a-FP-PhON**	0.23027	1.50735	0.1168	0.1241	7.57	6.18	25.1	34,000	23,300

The order parameter ⟨*P*_2_⟩ varies from ∼0.08 to 0.20, primarily depending on the μ* of PPH-phores. Notably, PPH-phores with the highest μ* and ⟨*P*_2_⟩, such as **F1-TCF-PhON** and **F2-TCF-PhON**, showed the lowest EO activity among the series. This is attributed to their reduced *β*_μ_^*^ values, which arise from a cyanine-like electronic structure.^2,13,36,37^

For the TCF-based series, replacing the **Cl** with the oxazolyl thioether group **PhON** as the *meso*-substitution generally enhances both the EO activity and *β*_μ_^*^ values while maintaining comparable or slightly increased *α*_33_^*^ for PPH-phores. It indicates that **PhON** substitution effectively improves the optical nonlinearities of PPH-phores without compromising the polarizability. Furthermore, the incorporation of stronger **FP** acceptor with the **F3** and **M1** donors significantly boosts the ⟨*P*_2_⟩, *α*_33_^*^, μ*, and *β*_μ_^*^ values of PPH-phores, over their TCF-based analogs.

Within the **F3** series, varying the *meso*-substituents (**PhON**, **SN**, **ONB**, and **SO2**) can fine-tune the *α*_33_^*^ and μ*. It demonstrates how such groups, with differing electronic, inductive, and steric effects, can tune linear and EO responses. Besides **M1a-FP-PhON**, **F3-FP-PhON** stands out for its highest *α*_33_^*^ values and the second highest *β*_μ_^*^, which correlate well with its large *r*_33_ value. Collectively, these comparisons underscore that the ROGM-derived molecular properties quantitatively capture subtle electronic-structure variations across these closely related PPH-phores, providing a clear pathway for property optimization.

## 4 Conclusions

This study successfully establishes a rigorous, integrated, experimental, and analytical workflow that directly bridges molecular engineering with bulk material performance in organic electro-optics. By combining ATR spectroscopy with Sellmeier dispersion analysis and Kuzyk’s two-component model, we demonstrated a reliable methodology for extracting critical molecular parameters, including the order parameter, permanent dipole moment, linear polarizability, and first hyperpolarizability of PPH-phores, directly from ATR spectroscopy of slab waveguide films. These critical parameters are otherwise difficult to obtain but essential for guiding the future development of high-performance organic EO materials.

The utility of this framework was validated through a comprehensive evaluation of 15 PPH-phores and 20 guest–host polymer systems. Notably, this systematic optimization led to the discovery of exceptional molecular properties, yielding an ultralarge dressed first hyperpolarizability and a record-high linear polarizability. These exceptional molecular properties directly translate into large EO coefficients of poled guest–host polymers.

Specifically, the ATR and ROGM analyses provide a quantitative link spanning chromophore structure, orientational order, microscopic response, and macroscopic EO response. The accuracy and reliability of the present study, and of our recent closely related work, rely critically on the availability of high-precision optical instrumentation, high-performance poled EO polymer films, and robust computational tools for data analysis. Notably, our determination of the dipole moments for 15 PPH-phores reveals μ* values substantially lower than those anticipated from DFT calculations and conventional design wisdom, implying that the magnitude of interchromophore dipole–dipole interactions at low to modest loading densities is correspondingly weaker than commonly assumed. The results indicate that maximizing the *r*_33_ values in slab waveguides requires the simultaneous optimization of the *β*_μ_^*^, *α*_33_^*^, and ⟨*P*_2_⟩, rather than focusing on any single parameter. Chromophores such as **M1a-FP-PhON** and **F3-FP-PhON**, which combine modest μ* and large *β*_μ_^*^ with relatively high orientational order, emerge as promising design archetypes for next-generation organic EO materials based on the PPH-phores.

Moreover, the internal consistency between the ATR-derived microscopic parameters and the measured *r*_33_ values validates the use of ATR–ROGM framework as a high-throughput screening methodology for new guest–host combinations. It can potentially reduce the necessity for full device fabrication during early stage materials discovery. By providing both macroscopic EO activity and mechanistic insight into how changes in molecular structure influence the orientation and bulk response, this offers a clear path to guide rational synthetic modifications aimed at further enhancing EO performance in poled polymer systems.

## Supplementary Material



## Notes

Supplementary note, analytical equations, ATR spectra, and summary of molecular and materials properties (PDF)
