# Supplementary material for: ATR Analysis: Quantifying Molecular and Bulk Responses in Guest–Host Poled Polymers with Push–Pull Heptamethine Chromophores
Source: J Phys Chem B. 2026 Aug 29;130(36):9256–67. doi: 10.1021/acs.jpcb.6c03666 (PMC13573552; doi:10.1021/acs.jpcb.6c03666)
Supplement: Supplementary file 1 [file jp6c03666_si_001.pdf]

Supporting Information

ATR Analysis: Quantifying Molecular and Bulk Responses in Guest-Host Poled Polymers with Push-Pull Heptamethine Chromophores

Danning Lyu,[1,2] Di Zhang,[1,2,3] and Jingdong Luo[1,2]**

[1] Department of Chemistry, City University of Hong Kong, Hong Kong SAR, China

[2] Shenzhen Research Institute, City University of Hong Kong, Shenzhen 518057, China

[3] State Key Laboratory of Flexible Electronics (LoFE) & Institute of Advanced Materials (IAM), School of Flexible Electronics (Future Technologies), Nanjing Tech University (NanjingTech), Nanjing 211816, China

*Corresponding author. E-mail: dzhang44-c@my.cityu.edu.hk; jingdluo@cityu.edu.hk

Table of Contents

Supplementary Note 1 .... S2

Determination of the μ* and <cos$^3\theta$> of poled polymer films from <P_2> S3

DC-modulated ATR mode shift under the positive and negative bias S4

Loading-density-dependent properties of poled guest-host polymers S5

References S6

Supplementary Note 1

Calculating the order parameter of poled films from the poling-induced optical birefringences

A Sellmeier fit of refractive index is carried out based on refractive index at 1.3 µm and 1.54 µm, by assuming that the refractive index (n) is contributed by a non-resonant background (n_0) and a two-level charge transfer resonance at frequency v_0 in cm^{-1}.[1,2]

$$n = n_0 + \frac{A}{v_0^2 - v^2} \qquad \text{(Equation S1)}$$

where n and v are the refractive indices of unpoled films and the frequencies at 1.3 µm and 1.54 µm, respectively. The v_0 is the frequency of chromophore/polymer films at the maximum absorption wavelength. The order parameter $\langle P_2 \rangle$ for poled films is given by

$$\langle P_2 \rangle = \frac{n_{\mathrm{TM}} - n_{\mathrm{TE}}}{n_{\mathrm{TM}} + 2n_{\mathrm{TE}} - 3n_0} \qquad \text{(Equation S2)}$$

The n_{TM} and n_{TE} are the ATR-measured refractive indices of poled films in transverse magnetic (TM) and transverse electric (TE) polarization, respectively.

Determination of the μ* and <cos$^3\theta$> of poled polymer films from <P_2>

From the rigid oriented gas model (ROGM) for poled polymers containing dipolar chromophores, the order parameter $\langle P_2 \rangle$ and the polar order parameter $\langle \cos^3\theta \rangle$ of poled films are the second and third Legendre polynomials as a function of $u = \mu^* E_{\mathrm{p}}/(kT)$, respectively, as shown in Figure S1.

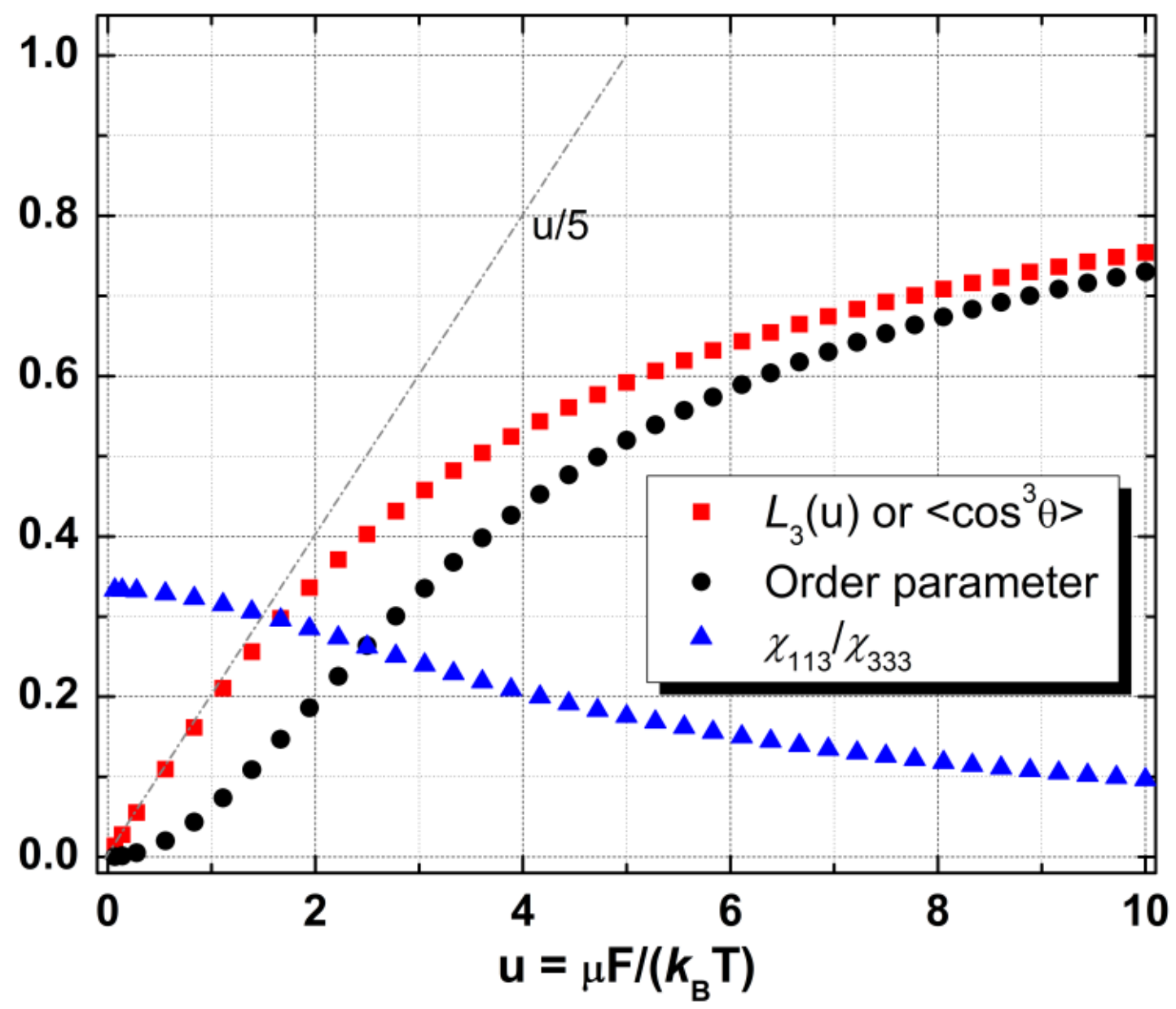

Figure S1. The order parameter and third Legendre polynomial as a function of u, where u = μF/kT. Reproduced with permission from Burland, D. M.; Miller, R. D.; Walsh, C. A., Second-order nonlinearity in poled-polymer systems, *Chem. Rev.*, 1994, *94*: 31-75. Copyright (1994) American Chemical Society.

DC-modulated ATR mode shift under the positive and negative bias

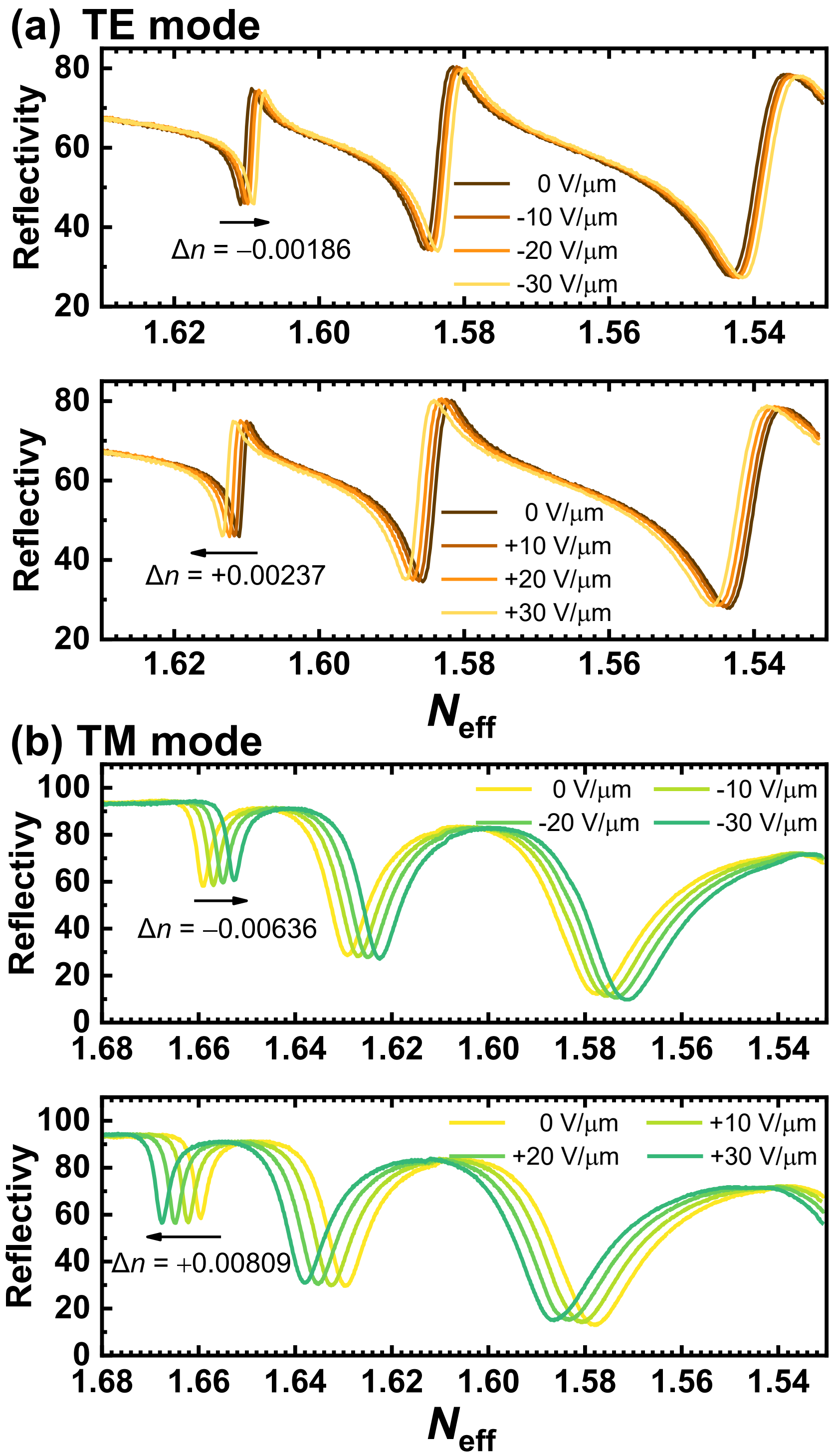

Figure S2. DC-modulated TE and TM mode shift of poled **M1a-FP-PhON**/P(S-*co*-MMA)/*N*1.30 film under the positive and negative voltages. The film thickness is 3.52 μm, and the wavelength is 1.3 μm.

Loading-density-dependent properties of poled guest-host polymers

Table S1. Optical, EO, and effective molecular properties of **M1a-FP-PhON**/P(S-*co*-MMA) films at 1.3 μm and 1.54 μm as a function of N.

N (cm^{-3})	E_p (V/μm)[b]	Unpoled films		Poled films		Sellmeier fitting constants		<P_2>		α_{33}* (10^{-22} cm^3)		μ* (Debye)		$-r_{33}$ (pm/V), DC		$-\beta_\mu$* (10^{-30} esu)	
		n_{TE}, n_{TM} at 1.3 μm	n_{TE}, n_{TM} at 1.54 μm	n_{TE}, n_{TM} at 1.3 μm	n_{TE}, n_{TM} at 1.54 μm	A	n_0	1.3 μm	1.54 μm	1.3 μm	1.54 μm	1.3 μm	1.54 μm	1.3 μm	1.54 μm	1.3 μm	1.54 μm
0.7×10^{20}	93.6	1.58603, 1.58614	1.57377, 1.57246	1.57974, 1.60313	1.56880, 1.58704	0.12286	1.51519	0.1105	0.1040	7.50	6.12	23.7	22.9	70.2	51.2	30,600	22,100
1.0×10^{20}	105.8	1.61179, 1.61144	1.59482, 1.59464	1.60336, 1.63922	1.58825, 1.61325	0.16561	1.51697	0.1257	0.1076	7.07	5.78	22.7	20.7	100.6	69.8	31,500	22,200
1.3×10^{20} [a]	94.7	1.63885, 1.63724	1.61658, 1.61225	1.62925, 1.67514	1.60790, 1.64877	0.23027	1.50735	0.1168	0.1241	7.57	6.18	24.2	25.1	124.6	94.0	34,000	23,300
1.5×10^{20}	98.1	1.65895, 1.65799	1.63196, 1.63353	1.64676, 1.70257	1.62073, 1.66035	0.26279	1.51027	0.1263	0.1113	7.48	6.11	24.5	22.7	117.6	82.3	28,500	19,200
1.7×10^{20}	103.9	1.67810, 1.67649	1.64726, 1.65029	1.66614, 1.71993	1.63631, 1.68619	0.29752	1.51061	0.1094	0.1225	7.47	6.11	21.2	22.7	113.3	61.6	27,100	12,900

Note: [a]The results differ slightly from the values reported for the poled films in Table 1 due to the use of different poling conditions for this formulation; [b]E_p was calculated from the poled film, which experienced a minor change in thickness after the poling process.

References

[1] Page, R. H.; Jurich, M. C.; Reck, B.; Sen, A.; Twieg, R. J.; Swalen, J. D.; Bjorklund, G. C.; Willson, C. G. Electrochromic and optical waveguide studies of corona-poled electro-optic polymer films. *J. Opt. Soc. Am. B*, **1990**, 7, 1239-1250.

[2] Burland, D. M.; Miller, R. D.; Walsh, C. A. Second-order nonlinearity in poled-polymer systems. *Chem. Rev.*, **1994**, *94*, 31-75.
